# Influence of copper on expression of *nirS*,* norB* and *nosZ* and the transcription and activity of NIR, NOR and N_2_ OR in the denitrifying soil bacteria *Pseudomonas stutzeri*

**DOI:** 10.1111/1751-7915.12352

**Published:** 2016-03-02

**Authors:** Amanda Black, Pei‐Chun L. Hsu, Kelly E. Hamonts, Tim J. Clough, Leo M. Condron

**Affiliations:** ^1^ Bio Protection Research CentreLincoln UniversityPO Box 85084LincolnChristchurch7647New Zealand; ^2^ Hawkesbury Institute for the EnvironmentUniversity of Western SydneyLocked Bag 1797PenrithNSW2751Australia; ^3^ Faculty of Agriculture and Life SciencesLincoln UniversityPO Box 85084LincolnChristchurch7647New Zealand

## Abstract

Reduction of the potent greenhouse gas nitrous oxide (N_2_O) occurs in soil environments by the action of denitrifying bacteria possessing nitrous oxide reductase (N_2_ OR), a dimeric copper (Cu)‐dependent enzyme producing environmentally benign dinitrogen (N_2_). We examined the effects of increasing Cu concentrations on the transcription and activity of nitrite reductase (NIR), nitric oxide reductase (NOR) and N_2_ OR in *Pseudomonas stutzeri* grown anaerobically in solution over a 10‐day period. Gas samples were taken on a daily basis and after 6 days, bacterial RNA was recovered to determine the expression of *nirS*,* norB* and *nosZ* encoding NIR, NOR and N_2_ OR respectively. Results revealed that 0.05 mM Cu caused maximum conversion of N_2_O to N_2_ via bacterial reduction of N_2_O. As soluble Cu generally makes up less than 0.001% of total soil Cu, extrapolation of 0.05 mg l^−l^ soluble Cu would require soils to have a total concentration of Cu in the range of, 150–200 μg g^−1^ to maximize the proportion of N_2_O reduced to N_2_. Given that many intensively farmed agricultural soils are deficient in Cu in terms of plant nutrition, providing a sufficient concentration of biologically accessible Cu could provide a potentially useful microbial‐based strategy of reducing agricultural N_2_O emissions.

## Introduction

Since the industrial revolution, global agricultural intensification and the use of artificial fertilizers has increased the amount of reactive nitrogen (N) in the natural environment by an order of magnitude (Galloway *et al.,* 2003; Richardson *et al*., 2009; Taylor and Townsend, 2010). This has resulted in a reorganization of the global N cycle causing a number of environmental problems including increased nitrous oxide (N_2_O) emissions (Taylor and Townsend, 2010; Magalhaes *et al*., 2011).

**Figure 1:**
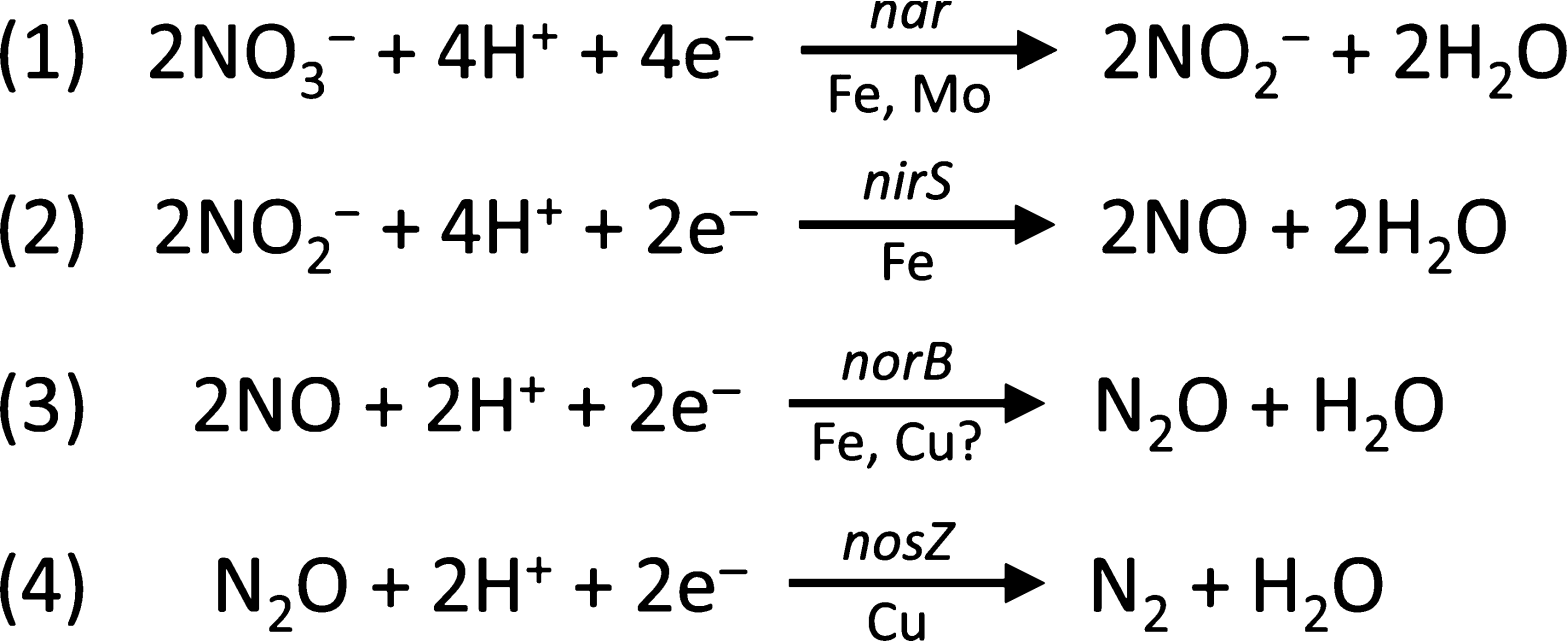
Nitrate is reduced to nitrogen gas under anaerobic condition via the denitrification process of *Pseudomonas stutzeri*. Arrows indicate an operon or gene required for each reaction to occur with metal cofactor requisite for the enzyme complex.

Nitrous oxide accounts for ~10% of the total greenhouse gas emissions and is produced as a by‐product of bacterial and fungal respiration pathways in soil. Both denitrification and nitrification respiratory pathways emit N_2_O with rates increasing with the addition of N fertilizer (Taylor and Townsend, 2010; Magalhaes *et al*., 2011). Denitrification occurs when oxygen (O_2_) is in limited supply and bacteria with denitrifying capability can switch to respiring nitrate (NO_3_^−^), converting NO_3_^−^ to nitrite (NO_2_^−^) and the gases nitric oxide (NO) and N_2_O and finally dinitrogen (N_2_) (Fig. 1). This process requires four enzymes to sequentially reduce NO_3_^−^ to N_2_ with each of these enzymes requiring a redox metal cofactor (Fig. 1). Denitrifying soil bacteria such as *Pseudomonas stutzeri* generate N_2_O via the reduction of NO, an endogenous cytotoxin, by reducing N_2_O via the enzyme nitric oxide reductase (NOR), hence bacteria deficient in NOR cannot grow through denitrification (Zumft, 2005a,b). Because so much N_2_O is produced from soils carrying out bacterial denitrification, it implies that the bacterial enzyme nitrous oxide reductase (N_2_OR), or the bacterial population as a whole, do not always carry out the reduction of N_2_O to N_2_ efficiently or in synchrony with pathways upstream (i.e. nitrifier‐denitrification) (Richardson *et al*., 2009). Thus, managing N_2_O emissions requires consideration of the factors affecting the production of N_2_O at both the molecular and soil microbial ecology levels.

Nitric oxide reductase is a key enzyme in the production of N_2_O and is responsible for catalysing the reduction of NO to N_2_O (Fig. 1, equation 3). Under intensive pasture management, N_2_O is the predominant end‐product during the denitrification process, which is a main contributor to global N_2_O emissions through the agricultural nitrogen cycle (Mosier *et al*., 1998). The structure of NOR has been well studied and is known to be a member of the haem‐copper oxidase superfamily (Zumft *et al*., 1994). The homology analogue of this protein taken from *Paracoccus denitrificans*,* Pseudomonas aeruginosa*,* Bacillus azotoformans* and *Ralstonia eutropha* were also comprehensively characterized (DeBoer *et al*., 1996; Pohlmann *et al*., 2000; Suharti *et al*., 2001) *P. aeruginosa*. NOR is expressed by *P. stutzeri* under anaerobic conditions (Korner, 1993) and is found exhibiting as a complex consisting of several components including high‐spin cytochrome b, low‐spin Fe(III) haem centre and a cytochrome c subunit or short‐chain NOR acting as the electron donor at the active centre (Zumft, 2005a,b). Although the majorities of NORs reported consist of iron‐binding site, remarkable structural similarities was found between *cbb3* cytochrom oxidase and NOR which contains a high‐spin haem‐copper (CuB) active site (Vanderoost *et al*., 1994). Furthermore, there is emerging evidence that NOR from *Bacillus azotoformans* to be a hybrid between copper‐containing cytochrome oxidases and NOR found in Gram‐negative bacteria (Suharti *et al*., 2001; Al‐Attar and de Vries, 2015). Due to the similarities between *cbb3* cytochrom oxidase and NOR, and lack of crystallography structure evidence, it can be questionable whether NOR from *P. stutzeri* is also regulated by copper metal cofactor. Moreover, Cu‐dependency of NOR from *P. stutzeri* has not been investigated before.

The reduction of N_2_O to N_2_ is strongly exergonic (Fig. 1, equation 4) (∆G°’ = −339.5 kJ mol^−1^), and electron delocalization stabilizes the molecule and leads to an activation energy barrier of 250 kJ mol^−1^ (Tolman, 2010). However, N_2_OR, first identified in 1982 (Zumft and Matsubara, 1982), is the only known enzyme capable of reducing N_2_O to N_2_ during the denitrification process (Zumft and Kroneck, 2007). The N_2_OR enzyme is a Cu‐dependent enzyme with a three‐dimensional structure revealing a multi‐Cu sulfide centre Cu_z_ [4:Cu:2S] where the two‐electron reduction of N_2_O takes place (Richardson *et al*., 2009). The Cu requirement for the active dimeric form of N_2_OR requires the bacterium to have an adequate supply of Cu. An absence of Cu in some culture studies has resulted in a rise in N_2_O emissions (Granger and Ward, 2003). While a Cu‐deficient denitrifying bacterial community can still remain viable, it is likely that they will release much higher levels of N_2_O. Several studies have documented the central role of Cu in enzyme structure and function (Matsubara *et al*., 1982; Farrar *et al*., 1998; Zumft, 2005a,b; Pomowski *et al*., 2010); however, the effects of Cu on the expression of these genes *nirS*,* norB* and *nosZ* to increase Cu bioavailability have yet to be described. We hypothesize that denitrifying bacteria have the ability to sense Cu availability and adjust the synthesis of N_2_OR in response to environmental conditions. In this study, we examine the effects of increasing Cu concentrations on the expression and activity of nitrite reductase (NIR), NOR and N_2_OR in *P. stutzeri* and the potential implications for molecular and stoichiometric influences on N_2_O emissions from agricultural soils.

## Results and discussion

### The effect of increasing copper concentrations on nitrous oxide accumulation and conversion into dinitrogen in anaerobic cultures of *Pseudomonas stutzeri*

**Figure 2:**
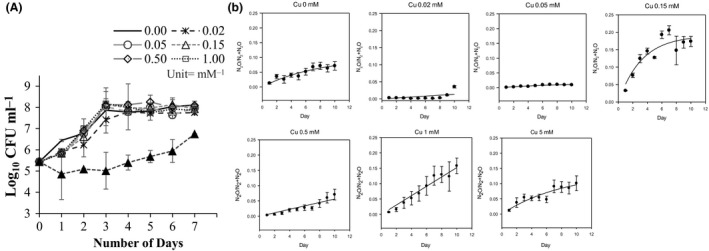
Growth curve of *Pseudomonas stutzeri* over the course of 7 days (A). Mean N_2_O:(N_2_+N_2_O) ratio of each copper treatment 0, 0.02, 0.05, 0.15, 0.5, 1, 5 and 20 mM over 10‐day period (B). Standard error of the mean (SEM) are represented as error bars.

**Figure 3:**
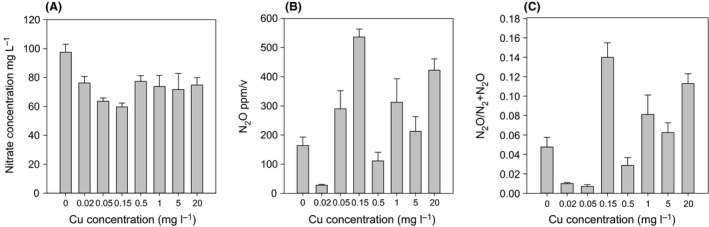
Mean nitrate (A) and N_2_O (B) concentrations after 10 days and mean N_2_O:(N_2_+N_2_O) ratio (C). Standard error of the mean (SEM) are represented as error bars. Groups with different letters differ significantly at the 5% level.

Growth of *P. stutzeri* from anaerobic cultures over 7‐day period showed no significant difference throughout all Cu concentration, except 5.00 and 20.00 mM of Cu (Fig. 1A), and the growth reached stationery phase at day 3 with ≤ 1.00 mM of Cu. Colony counts of *P. stutzeri* cultures containing 5.00 mM Cu were significantly reduced compared with those in Cu concentrations < 5.00 mM with a delay in growth after day 3 (Fig. 1A). This could indicate the onset of toxicological effects, although denitrification was occurring at relatively high rate (Fig. 2B). As expected, given *P. stutzeri* the highest concentration of Cu (20.00 mM) inhibited the growth completely (result not shown). Daily measurements revealed production of N_2_O and subsequent reduction to N_2_ varied significantly with changes in Cu concentrations (Fig. 2B). Interestingly, the Cu‐deficient broth (control, 0.00 mM) did not produce the highest concentrations of N_2_O compared with the Cu‐containing treatments as has been suggested in a previous study to be the most likely outcome (Granger and Ward, 2003). Furthermore, a comparison between the total amount of N_2_ produced per Cu treatment revealed no significant differences. In fact, the highest amount of N_2_O produced was observed in the culture containing 0.15 mM, which corresponded to the amount of NO_3_^−^ consumed (Fig. 3A and B). The proportion of N_2_O to N_2_O + N_2_ in the headspace differed significantly (*P* < 0.001) between Cu treatments, with the lowest proportion of N_2_O to total N measured in the treatments containing 0.02 and 0.05 mM of Cu (Fig. 3C). Over the 10‐day sampling period (apart from 0.50 mM Cu treatment), treatments ≥ 0.15 mM Cu resulted in a significantly higher (*P* < 0.001) total yield of N_2_O than N_2_. Studies that have investigated the effect of Cu addition on the conversion rate of N_2_O to N_2_ concluded that the absence of Cu resulted in an accumulation of N_2_O (Granger and Ward, 2003; Manconi *et al*., 2006; Felgate *et al*., 2012). However in these studies, the Cu concentration at which N_2_OR maintained an optimum activity and where the lowest proportion of N_2_O to N_2_ levels were generated, were not evaluated in further detail. Additionally, the presence of sulfide, especially H_2_S in a Cu‐deficient environment has been known to affect the reduction of N_2_O to N_2_ (Manconi *et al*., 2006; Pan *et al*., 2013). Given that in our study, S_2_^−^ and H_2_S could have been generated during anaerobic incubation of the broth from CuSO_4_ (source of the Cu) and cysteine, this may have also contributed to the accumulation of N_2_O.

### The effect of copper availability on the expression of *nirS*,* norB* and *nosZ*

**Figure 4:**
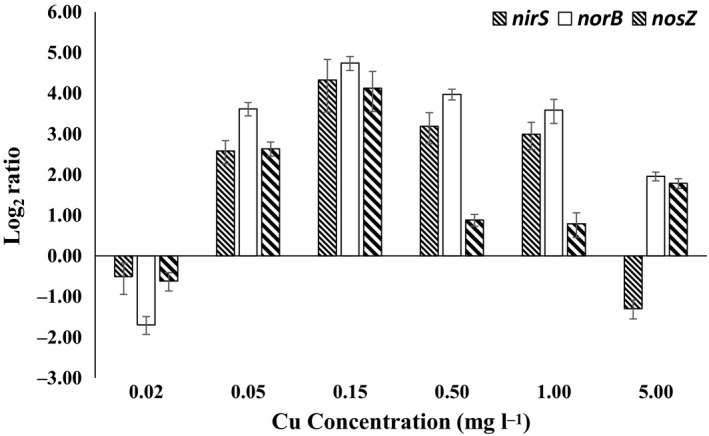
Expression ratios of the *Pseudomonas stutzeri nirS*,* norB* and *nosZ* at 5 days post‐inoculation. Expression ratios were calculated and normalized against reference genes *fdxA, ropD* and *gyrB*. Expression ratios are the difference in gene expression of *Pseudomonas stutzeri* cultured in basal salt solution with different copper concentration relative to the RNA expression in culture incubated without the presence of copper. Error bars are the SEM for all sample replicates.

From the Cu treatments it emerges that a Cu concentration of 0.15 mM is the level at which *nirS*,* norB* and *nosZ* expression are at their highest (Fig. 4), although N_2_O level and the mean N_2_O:(N_2_+N_2_O) ratio was at the highest of all Cu concentration given. Our results show inhibition of *nosZ* transcription from a Cu concentration of 0.50 mM onwards. Interestingly, even though the *nirS* enzyme contains a haem‐iron cofactor, it appears the transcription of *nirS* is responsive to changes in Cu concentrations (Fig. 4). Furthermore, *norB* expression was also declined with increased Cu concentration. Similarly, in a previous study conducted by Magalhães *et al*. (2011), a pronounced inhibition by Cu on the transcription of *nosZ* and *nirS* was detected. In the treatment containing 0.15 mM of Cu, the N_2_O:(N_2_+N_2_O) ratio was elevated as well as the observation of comparatively high levels of denitrifying genes expressed. This suggests that there could exist an alternative pathway for *P. stutzeri* to synthesize N_2_O at higher levels of Cu; thus, the level of N_2_O produced due to NOR activity could be overestimated in this study. X‐ray crystallographic structures of the periplasmic membrane protein *cbb3* cytochrome oxidase of *P. stutzeri* revealed a high‐spin haem‐copper (CuB) binuclear centre of which the catalytic active site was also found reducing NO to N_2_O during denitrification process via the proton pathway through K‐channel (Forte *et al*., 2001; Buschmann *et al*., 2010). Increased Cu concentration might also contribute in elevating the N_2_O level via NO reduction by *cbb3* cytochrome oxidase in this study. Therefore, expression of genes involved in *cbb3* cytochrome oxidase (*cco*QNOS) should further be investigated. This may provide a more detailed insight of N_2_O formation regulated by Cu from an alternative pathway. The enzyme N_2_OR is considered as the only protein catalysed by Cu due to the multi‐Cu‐sulfide redox centres (Pomowski *et al*., 2010). Surprisingly, expression of *nosZ* did not respond to the incensement of Cu concentration in this study, instead, the transcriptional level of *nosZ* decreased tremendously at 0.50 mM of Cu onward (Fig. 4). This suggested high level of Cu could be displaying inhibitory effect. This result differs with a study conducted by Felgate *et al*. (2012) where transcription of *nosZ* of *P. denitrificans* was found upregulated at high Cu concentration (13 μmol l^−1^ which is equivalent to 2 mM).

Felgate *et al*. (2012) tested both pure (*P. denitrificans*) and mixed denitrifying cultures using 13.00 μM and 0.50 μM Cu in excess and limiting NO_3_^−^ and C availability to investigate the production of N_2_O and the accumulation of intermediate products, namely NO_2_^−^. Although the Cu concentrations used were an order of magnitude lower than the lowest concentrations used in this study and atypical of bioavailable Cu concentrations found in agricultural soils (Black *et al*., 2011), results demonstrated that if sufficient NO_3_^−^ was present when Cu was depleted, *P. denitrificans* would maintain biomass, but release N_2_O at a rate > 1000 times the rate of the corresponding Cu replete cultures. It was also noted that the N_2_O electron acceptor lost under Cu‐depleted conditions was compensated for by increased (≈20%) consumption of NO_3_^−^ compared with cultures replete in Cu. This observation of N_2_O being produced more quickly but consumed more slowly is mirrored when comparing the control and 0.02 mM Cu treatment concentration (Fig. 2A and B), although nitrate consumption did not increase. The N_2_O:(N_2_O+N_2_) trend observed in this study reflected observations made in a denitrifying community sourced from river sediments which were enriched in Cu due to agriculture run‐off (Magalhaes *et al*., 2011). The suggested reason for the observed pattern was the different sensitivities of each of the enzymes that catalyse the first three steps of denitrification (NO_3_^−^ → NO_2_^−^, NO_2_^−^ →NO^−^ and NO^−^ → N_2_O) (Magalhaes *et al*., 2011). Similarly, the authors found that denitrifcation rates were highly affected by Cu concentrations, moreover they also observed a decrease in the diversity of *nirK, nirS* and *nosZ* and the corresponding transcribed enzymes with increasing Cu.

Results from this study suggest that Cu bioavailability can influence the expression and activity of N_2_OR and NIR, as well as the growth rates of *P. stutzeri* and imply that it may be possible to use stoichiometry to manage N_2_O emissions from agricultural soils. This approach has been previously suggested as a possible strategy to mitigate N_2_O emissions by providing an adequate source of essential micronutrients particularly Cu, Mo and Fe for redox reactions (Richardson *et al*., 2009). Although the reduction of N_2_O to N_2_ is not energetically favoured under optimal conditions, this reduction does occur at no loss of energy requirements to the bacteria. Moreover, our results confirm that there indeed exists an optimal Cu concentration threshold for *P. stutzeri* with respect to maximizing N_2_O consumption.

## Conclusion

In summary, our findings do support a key role for Cu in the regulation of N_2_O emissions, by demonstrating a Cu concentration gradient effect in the production and consumption of N_2_O. While total soil Cu is still the most common soil measure of bioavailability, bioavailable Cu is typically < 0.001% of total soil Cu (Bolan *et al*., 2003; McLaren *et al*., 2010; Black *et al*., 2011). Thus, values of 0.02–0.15 Cu mM used in this study are more realistic representations of biologically available Cu in agricultural soil environments to consider for potential management strategies of N_2_O emissions (Richardson *et al*., 2009). Extrapolating the Cu concentrations used in this study into an agricultural setting imply in some situations, current soil levels of Cu maybe deficient to allow this enzymatic pathway to operate at an optimal level. Calculating back from total soil Cu concentrations to biologically available Cu (0.05–0.10 mM Cu in solution) that is required to attain maximum conversion of N_2_O to N_2_, equates to a total amount of soil Cu in the range 150–200 μg g^−1^ (Black *et al*., 2011). Soils containing higher concentrations of Cu may result in inhibition of denitrification via a shift in denitrifier community composition (Manconi *et al*., 2006; Taylor and Townsend, 2010; Felgate *et al*., 2012) and correspondingly, Cu‐deficient soils may result in N_2_O production exceeding N_2_O conversion to N_2_.

Using one species of denitrifying bacteria in a simple basal salt solution has provided some insight into the effects of increasing Cu bioavailability on the transcription and activity of N_2_OR and NIR to reduce N_2_O to N_2_. We observed that adequate bioavailable Cu concentrations (0.15 mM) resulted in the greatest transcription of the *nirS*,* norB* and *nosZ*, which is not the optimal consumption of N_2_O to produce N_2_. However, the level of N_2_O may be overestimated due to another possible alternative pathway of NO reduction by *cbb3* cytochrome oxidase. Based on our experience with measuring amounts of bioavailable soil Cu, extrapolation of this soluble Cu concentration to an agricultural soil environment equates to total soil Cu concentrations in the range of 150–200 μg g^−1^. Furthermore, a majority of soils globally under intensive agriculture, are now considered too deficient in Cu to complete the last stages of the denitrification process to N_2_ (Skrbic and Durisic‐Mladenovic, 2010). Therefore, in addition to other mitigation methods, supplying adequate Cu would contribute to an overall effective management of N_2_O emissions in increasing N intensification systems. Future studies will need to investigate if similar Cu concentrations may apply to other soil denitrifying bacteria, including investigating the possibility of adopting a Cu strategy for whole soil microbial communities. However, any strategy employed would have to consider any potential toxicity issues for grazing animals.

## Experimental procedures

### Cultivation conditions of *P*. *stutzeri* (ATCC 17588)

The denitrifying bacteria *P. stutzeri* (ATCC 17588) was chosen due to the presence of *nirS* (Fe‐cofactor) instead of *nirK* (Cu‐cofactor). Bacteria were first grown in cultures under aerobic conditions. The basal salt solution of the aerobic medium comprised the following (mM): 1.5 KH_2_PO_4_, 5.6 NH_4_Cl, 0.5 MgCl_2_·6H_2_O, 0.7 CaCl_2_·2H_2_O, 0.4 MgSO_4_·7H_2_O and 30 KNO_3_. The solution was autoclaved and after cooling 99% D‐Na lactate (Sigma Aldrich, St Louis, Missouri, United States) was added to a final concentration of 31 mM in 100 ml volume (Johnsson *et al*., 2006). The aerobic culture was grown in 100 ml solution contained per 250 ml Erlenmeyer flasks for 4 days before 1 ml of culture‐containing solution, containing approximately 10^6^ bacterial cells, was transferred into the anaerobic Cu medium experiment.

The basal salt solution of the anaerobic medium comprised (mM) 0.06 KH_2_PO_4_, 5.6 NH_4_Cl, 0.5 MgCl_2_·6H_2_O, 0.8 0.7 CaCl_2_·2H_2_O, 0.4 MgSO_4_·7H_2_O and 11 mM of ^15^N‐enriched KNO_3_ (10 atom%). The pH was adjusted to 8 using 1 M NaOH, with sufficient KH_2_PO_4_ in solution to provide buffering capacity (Johnsson *et al*., 2006). Cu sulfate (CuSO_4_) was added to each gas‐tight medical boston bottle at concentrations equivalent to 0.00, 0.02, 0.05, 0.15, 0.50, 1.00, 5.00, 20.00 mM of Cu. The basal solution (100 ml per bottle) with Cu treatments was autoclaved and 99% D‐Na lactate and cysteine‐HCl (Sigma Aldrich) were added to produce final concentrations of 4.40 and 4.50 mM respectively. Copper treatments were performed in triplicate and each treatment replicate was inoculated with 1 ml of 10^6^ bacterial cells before being fitted with gas‐tight Teflon^®^ septa and purged using 99.99% Argon (Ar) (remaining 0.01% comprising CO_2_ < 5 ppm, N_2_ < 25 μl l^−1^, O_2_ < 10 μl l^−1^, H_2_O < 10 μl l^−1^, CO, C_2_H_6_, CH_4_ < 1 μl l^−1^) to create an anaerobic environment with minimum N_2_. Non‐inoculated controls were run simultaneously. The total headspace volume of each bottle was 150 ml. Cultures were grown in a pressurized Ar atmosphere at room temperature (23°C) and were agitated on an orbital shaker for 10 days. Colony‐forming units were used to estimate the number of viable cells for each replicated treatment each day using 10‐fold dilution (up to 10^−9^) with 100 μl of diluted cells plated out onto solidified Luria–Bertani agar (Oxoid Thermo Fisher Scientific Inc. Waltham, MA, United States) and incubated for 3 days at room temperature (24°C) for 3 days.

### Gas sampling and analyses

Gas samples were taken on a daily basis for 10 days (2 days after inoculation) where 1 ml of headspace was extracted and added to 11 ml of helium (99.99% pure) contained in 12 ml glass Exetainers^®^. Sample volume removed was replaced with 1 ml of Ar. Samples were analysed for Ar, O_2_, N_2_, N_2_O and ^15^N using a Continuous Flow Isotope Ratio Mass Spectrometer (PDZ Europa TGII/20‐20). The concentration of ^15^N‐N_2_ was below the limit of quantification and thus not reported. Accuracies of Cu concentrations per treatment were verified at ± 10% at the end of the experiment using inductively coupled plasma optical emissions spectrophotometer (ICP‐OES).

### Anion and cation analyses

After 10 days, 10 ml of the growth medium was filtered using a 0.22 μm cellulose filter (DISMIC, Advantec, Tokyo, Japan) and analysed for NO_2_^−^ and NO_3_^−^ concentrations using a Dionex DX‐120 Suppressed Ion Exchange Chromatograph (Dionex Corporation, Sunnyvale, CA, USA). Copper concentrations were confirmed using an ICP‐OES Varian 720‐ES fitted with a SPS‐3 auto‐sampler and ultrasonic nebulizer.

### Expression of *nirS*,* norB* and *nosZ*

Bacterial RNA was extracted from the centrifuged bacterial pellet (20 000 r.p.m. for 10 min at 4°C) at day 5 using Roche High Pure RNA Isolation Kit (Cat. No. 11 828 665 001; Basel, Switzerland) according to the manufacturer's specifications. The RNA was treated twice with RNase‐free DNase I recombinant (Roche) to remove any genomic DNA contamination. The RNA was then stored in RNA*later* (Invitrogen, Boston, MA, USA) at −80°C before cDNA synthesis using Superscript III Supermix First Strand cDNA Synthesis Kit (Invitrogen) according to the manufacturer's instructions. The quality of the extracted RNA and cDNA was confirmed by gel electrophoresis, and yields of RNA and cDNA were measured with a NanoDrop ND‐1000 spectrophotometer (NanoDrop Technologies, Wilmington, DE, USA).

Transcripts of *nirS*,* norB* and *nosZ* encoding the cytochrome *cd*_*1*_ haem NIR, NOR and N_2_OR, respectively, were quantified by qRT‐PCR. Specific primers for three reference genes *fdxA*,* ropD* and *gyrB*, and one denitrification gene, *norB,* were designed from *Pseudomonas stutzeri* ATCC 17588 genome sequence (accession: PRJNA68131) using the web‐based program Primer‐BLAST (Ye *et al*., 2012) to generate ~200 bp DNA sequences (Table 1). The cDNA products were amplified with an ABI Prism 7000 (Applied Biosystems, Mulgrave Australia) using SensiFAST^™^ SYBR^®^ Hi‐ROX Kit (Bioline, London, UK) according to supplier's instructions. Quantitative RT‐PCR was performed with an initial denaturation at 95°C for 2 min, followed by 40 cycles of 95°C for 5 s, annealing at 60°C for 10 s and extension at 72°C for 20 s, with exception to the *nirS* which was amplified with an extension step for 30 s at 72°C.

**Table 1:** A list of oligonucleotide primers used in this study

Gene	Primer name	Sequences (5′–3′)	Fragment	Reference
*fdxA*	fdxA(F)	CCGGTGGACTGCTTCTACGA	208 bp	Present study
	fdxA(R)	CGGCCAGTGCATCCTTCTTC		
*gyrB*	gyrB(F)	GAATACCTCACCCAGTCGGC	198 bp	Present study
	gyrB(R)	CGTGACCCGAGGCAGATAGA		
*ropD*	ropD(F)	AGTACGATGCCCTGGTCGAG	198 bp	Present study
	ropD(R)	CGATGGCTTCGGCGTACTTG		
*nirS*	nirSCd3aF	AACGYSAAGGARACSGG	406 bp	Kandeler *et al*. (2006)
	nirSR3Cd	GASTTCGGRTGSGTCTTSAYGAA		
*norB*	norB(F)	CCATGCTCAAGGGTCGCAAG	197 bp	Present study
	norB(R)	CAGGACGAAGGCCAACATCG		
*nosZ*	nosZ1840F	CGCRACGGCAASAAGGTSMSSGT	185 bp	Henry *et al*. (2006)
	nosZ2090R	CAKRTGCAKSGCRTGGCAGAA		

Raw data were analysed based on Pfaffl *et al*. (2004) mathematical model to determine the relative quantitation of the target genes (*nirS*,* norB* and *nosZ*) that are normalized by three non‐regulated reference genes (*fdxA*,* ropD* and *gyrB*). High resolution melting analysis was performed to determine PCR integrity and primer dimers at the end of each run. Each qRT‐PCR efficiency is within the value between 1.60 and 2.10 and a *R* ^2^ value of > 0.980. The qRT‐PCR was performed in triplicate, and the means and standard errors were calculated.

### Statistical analysis

Statistical analysis on gas results, ion chromatography for broth chemistry and colony‐forming units were performed on log‐transformed data using one‐way ANOVA in GenStat (GenStat14; VSN International, Hemptstead UK). Non‐linear regression to evaluate the relationships between ratios of N_2_O:N_2_+N_2_O per treatment over the 10‐day period was performed in GenStat (GenStat 16.1; VSN International).
